# Outcomes of Spironolactone Withdrawal in Dilated Cardiomyopathy With Improved Ejection Fraction

**DOI:** 10.3389/fcvm.2021.725399

**Published:** 2021-09-16

**Authors:** Yanjia Chen, Zeping Qiu, Jie Jiang, Xiuxiu Su, Fanyi Huang, Jing Tang, Wei Jin

**Affiliations:** ^1^Department of Vascular and Cardiology, Ruijin Hospital, Shanghai Jiao Tong University School of Medicine, Shanghai, China; ^2^Institute of Cardiovascular Diseases, Shanghai Jiao Tong University School of Medicine, Shanghai, China; ^3^Department of Emergency Medicine, Ruijin Hospital, Shanghai Jiao Tong University School of Medicine, Shanghai, China

**Keywords:** heart failure with improved ejection fraction, spironolactone, dilated cardiomyopathy, withdrawal, heart failure management

## Abstract

**Background:** The feasibility of spironolactone withdrawal in dilated cardiomyopathy patients with improved ejection fraction remains unknown. This study sought to determine whether spironolactone can be withdrawn safely in this circumstance.

**Methods:** Consecutive patients with idiopathic dilated cardiomyopathy and prescribed spironolactone at discharge were included in this prospective, observational cohort using the Risk Evaluation and Management in Heart Failure Trial (NCT02998788) database. Those patients who experienced an absolute left ventricular ejection fraction (LVEF) improvement ≥10% and a second measurement of LVEF >40% would choose whether to continue spironolactone therapy and be included in final analysis. The primary endpoint was dilated cardiomyopathy relapse within 12 months, defined as a more than 10% reduction in LVEF, a 15% or greater increase in LVESVi, a 2-fold rise in NT-proBNP, or clinical signs of heart failure.

**Results:** Seventy patients achieved an ejection fraction improvement and were included in the final analysis, of whom 30 chose to continue spironolactone and 40 decided to withdraw. In primary endpoint analysis, 23 (58%) patients from the withdrawal group and 4 (13%) patients from the continuation group relapsed (relative risk for relapse: 4.31; 95% CI: 1.67–11.11; *p* < 0.001). Patients from the withdrawal group experienced more symptom aggravation than the continuation group. No secondary safety endpoint was recorded. Improvements in cardiac structure parameters were no longer observed after spironolactone withdrawal, while improvements persisted in continuation group.

**Conclusions:** Most dilated cardiomyopathy patients with improved ejection fraction will relapse after spironolactone withdrawal. These results should be weighed before spironolactone withdrawal was attempted.

## Introduction

Mineralocorticoid receptor antagonists (MRAs) are well-documented to reduce mortality and hospitalizations in patients with heart failure with reduced ejection fraction (HFrEF) (1, 2). Based on compelling clinical evidence, MRAs received a Class I recommendation for all symptomatic patients with left ventricular ejection fraction (LVEF) ≤ 35%, regardless of angiotensin-converting enzyme inhibitors or β-blockers (3–5). Although variable outcomes have been recorded in patients with dilated cardiomyopathy, the disease is a relatively benign process for most patients (6) and a clinical recovery reflected by LVEF improvement and left ventricle volume reduction can be achieved in around 40% patients after recommended therapies (7).

Following the symptom relief and cardiac function improvement, patients frequently questioned whether all heart failure medications are vital to continue indefinitely in concern of side-effects and inconvenience. Nowadays, practitioners have to act individually without sufficient evidence, weighing potential risks against uncertain benefits, and determined personalized MRA continuation strategy in asymptomatic heart failure patients with LVEF improved. Indeed, it is unknown whether patients with a diagnosis of HFrEF would benefit from MRA treatment continuation even after a clinical and imaging parameter of improved cardiac function. For certain patients who might have achieved permanent myocardial recovery, treatment continuation provide only disadvantage without additional benefits. Nevertheless, for patients only in a remission status, relapse could easily develop if treatment was withdrawn imprudently (8, 9). No sufficient evidence existed in our knowledge for decision of MRA treatment withdrawal in idiopathic dilated cardiomyopathy patients with improved ejection fraction. Consequently, no consensus among experts or clear recommendations in guidelines is available for now. Accordingly, we conducted a prospective observational study to explore the effects of MRA withdrawal in patients with clinical remission of dilated cardiomyopathy and improved LVEF.

## Methods

### Study Population and Clinical Care

For the current study, we consecutively screened 311 patients aged 18–89 years and admitted for newly diagnosed HFrEF at Ruijin Hospital, Shanghai Jiao Tong University School of Medicine between June 2016 and June 2017. Cases of newly diagnosed HFrEF were defined as patients with an LVEF lower than 40% and presented heart failure–associated symptoms and/or signs based on the 2016 ESC Heart Failure Guidelines, and patients with medical records of heart failure before were excluded. Only patients diagnosed with idiopathic dilated cardiomyopathy according to their laboratory tests, coronary angiogram, and cardiac magnetic resonance results by at least two experienced heart failure specialists independently and a prescription of spironolactone at discharge were included in further follow-up. Genetic sequencing was not required. All participants were regularly evaluated at office or on phone after discharge and received optimal pharmacological therapy up-titration and device treatment if indicated, as clinical practice guidelines recommended. Echocardiograms were performed at every office visit during follow-up. Patients presented with an improved ejection fraction would be deemed recovery and enter final study. In accordance with universal definition of heart failure (10), heart failure with improved ejection fraction (HFimpEF) was defined as follows: (1) a baseline LVEF <40%; (2) absolute LVEF improvement ≥10%; (3) a second measurement of LVEF >40%. Once a patient met the HFimpEF criteria, a cardiologist would thoroughly discuss with the patient if withdrawal or continuation of spironolactone was preferred, when maintaining other necessary heart failure medications, including but not limited to ACEI/ARB/ARNI and β-blocker. Each patient was provided with state-of-the-art clinical evidence about pharmacological treatment withdrawal and prognosis in DCM with improved ejection fraction during discussion. As long as no indication presented, the final decision of spironolactone continuation or not was solely made by the patient's own determination. Those ceased spironolactone treatment because of counter indications or side-effects, like hyperkalemia and gynecomastia, were excluded. All patients would be visited monthly after treatment decision executed, before a landmark analysis of endpoints was performed within 1 year after HFimpEF identification. If any sign of relapse occurred or indication for spironolactone re-emerged after discontinuation, the spironolactone treatment would be re-initiated immediately with other necessary interventions taken place. We recorded the time when LVEF improved as visit 1 (V1) and the final 1-year outcome analysis visit as visit 2 (V2) for better understanding.

### Data Collection

This single-center, open-label, prospective, and observational cohort study used de-identified individual data from the Risk Evaluation and Management in Heart Failure Trial (NCT02998788) database. The research was approved by the Institutional Review Board of Ruijin Hospital, Shanghai Jiao Tong University School of Medicine. All participants provided written informed consent.

### Clinical, Biochemical, and Echocardiographic Assessments

The following admission data were collected from electronic medical records: demographic data including age, gender, systolic blood pressure, body surface area (BSA), comorbidity, class of New York Heart Association (NYHA), N-terminal pro-brain natriuretic peptide (NT-proBNP), and echocardiographic parameters. Concentrations of NT-proBNP and echocardiography parameters were recorded at V1 and V2, respectively. The blood samples were collected in a quiet, air-conditioned room after overnight fasting and at least 20-min rest in supine position. Measurement of NT-proBNP was performed using a commercially available electrochemiluminescence immunoassay kit (Roche Diagnostics). BSA was calculated using the following formula: 0.0061 × height (cm) + 0.0128 × weight (kg) − 0.1529. Blood pressure was measured on the non-dominant arm in a seated position after a 10-min rest using an electronic blood pressure monitor (OMRON Model HEM-752 FUZZY; Omron Co., Dalian, China). Three measurements were taken at 1-min intervals, and the average value was used for analysis.

Transthoracic echocardiography was performed using a commercially available system (Vivid-I; GE Healthcare, Milwaukee, WI) with a 1.9–3.8-MHz phased-array transducer. Echocardiography was performed by a designated sonographer credentialed in cardiac ultrasound, who was not aware of the patient's treatment regimen. Two-dimensional (2D), pulsed-Doppler imaging was performed from standard parasternal and apical transducer positions with 2D frame rates of 60–100 frames/s. All data were stored digitally, and offline data analysis was performed (EchoPac, version 7; GE Healthcare) at the conclusion of the study by two cardiologists blinded to the study time point. The LVEF was calculated using the modified Simpson's biplane technique. The left ventricle (LV) length was measured in the apical four-chamber view, where LVEDD was LV end-diastolic diameter and LVESD was LV end-systolic diameter. LV end-diastolic volume (LVEDV) and LV end-systolic volume (LVESV) were recorded and indexed by BSA at each study time point. LV mass was estimated from M-mode measurements by the following formula: LV mass = 0.8 × 1.04 × [(LVEDD + IVST + LVPWT)^3^ − LVEDD]^3^ + 0.6, where IVST is the interventricular septal thickness and LVPWT is the LV posterior wall thickness. LV mass was indexed by BSA.

### Outcomes

The primary endpoint was relapse of dilated cardiomyopathy within 12 months, defined by meeting at least one of three criteria: (1) a more than 10% LVEF reduction or a more than 15% LVESVi increase in echocardiogram; (2) a 2-fold rise in V1 NT-proBNP concentration; (3) clinical signs and symptoms of heart failure adjudicated by clinicians. Secondary endpoints comprised a composite safety endpoint including cardiovascular mortality, major adverse cardiovascular events, and unplanned cardiovascular hospitalization.

### Statistical Analysis

Data are demonstrated as mean ± SD or median (interquartile range) for continuous variables, and frequency (percentages) for categorical ones. For continuous variables, normal distribution was evaluated using the Kolmogorov–Smirnov test. Skewed variables (e.g., NT-proBNP) were log-transformed to achieve a more normal distribution. Differences among groups were analyzed by Student's *t*-test or one-way ANOVA followed by *post hoc* Bonferroni test. Intergroup comparisons of categorical variables were performed using the χ^2^ test. Regression analysis of covariance was implemented to assess the effect of spironolactone withdrawal on changes in clinical parameters of HFimpEF patients over a 1-year follow-up period. All statistical analyses were performed using SPSS 23.0 for Windows (SPSS, Inc., Chicago, IL, USA). To conduct the subgroup analysis, R software (version 4.0.2; R Foundation for Statistical Computing, Vienna, Austria) was used. A two-tailed *p* value < 0.05 was considered statistically significant.

### Patient and Public Involvement Statement

This study was completed without patient involvement. Patients were not invited to comment on the study design or contribute to the writing or editing of this document.

## Results

### Patient Enrollment

**Figure 1:**
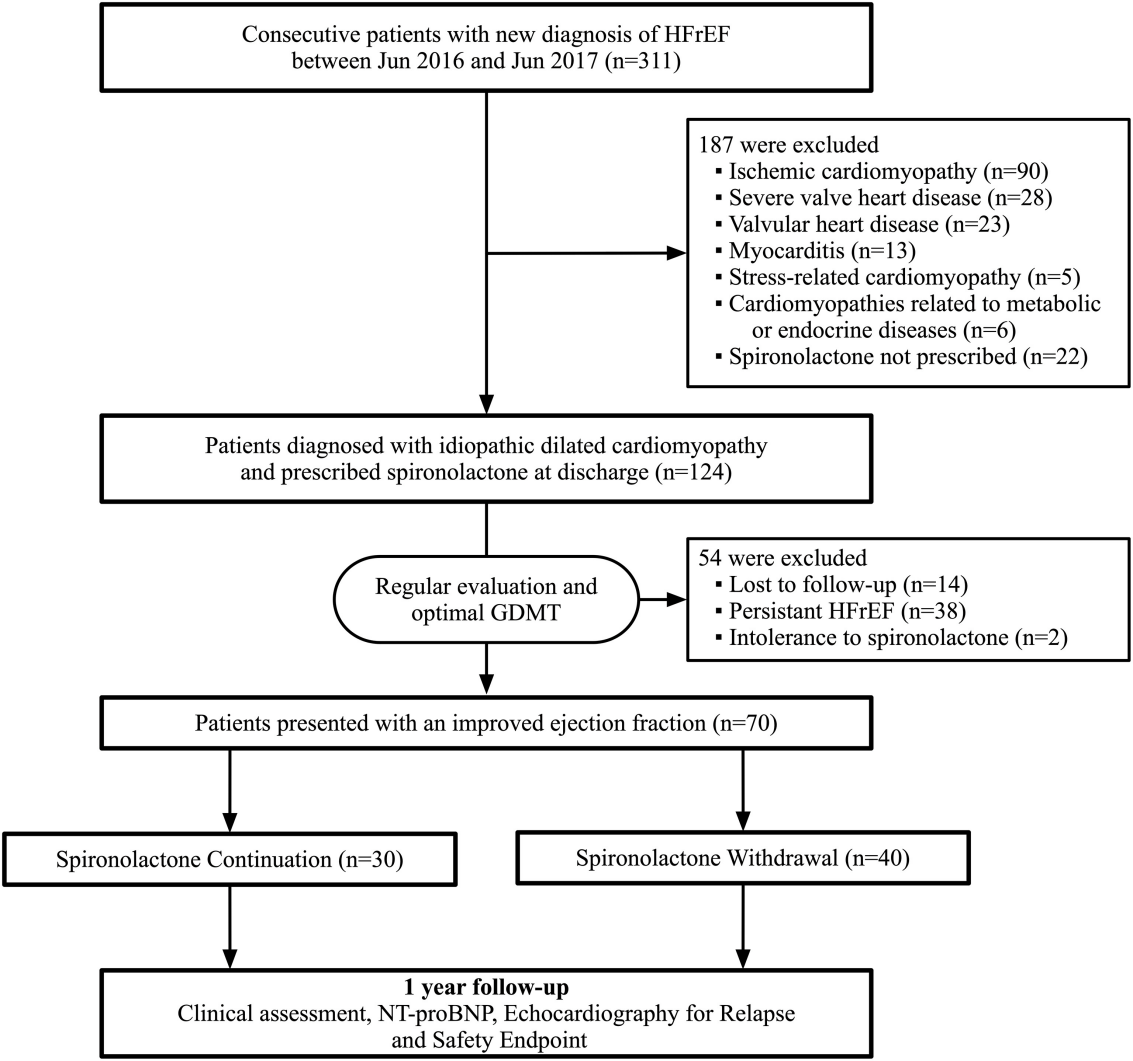
Flowchart of study profile. HFrEF, heart failure with reduced ejection fraction; GDMT, guideline-directed medical therapy; NT-proBNP, N-terminal pro-B-type natriuretic peptide.

Between June 2016 and June 2017, a total of 311 patients admitted for newly diagnosed HFrEF were screened during hospitalization. A total of 187 were excluded due to specific causes of heart failure, including 90 with ischemic cardiomyopathy, 28 with severe valvular disease, 23 with valvular heart disease, 13 with myocarditis, and 33 with other reasons. Moreover, 38 patients presented a persistent low ejection fraction during follow-up and 2 patients were excluded due to spironolactone cessation attributed to side-effects. Eventually, 70 patients were included in the final analysis according to our study population criteria. Within these 70 participants, 30 of them chose to continue spironolactone, while 40 patients decided to initiate withdrawal after ejection fraction improvement (Figure 1). The baseline characteristics at initial diagnosis were generally similar between the two groups (Supplementary Table 1).

### Baseline Characteristics at Recovery

Overall usage rate of guideline-recommended disease-modifying agents, including ACEI/ARB/ARNI and β-blocker, were 97.2 and 92.9%, respectively. Fewer patients in the withdrawal group were taking diuretics at discharge, but the difference was statistically insignificant. Average dose of spironolactone was 19.67 mg in the continuation group compared with 20.75 mg in those discontinued at V1. Among those who decided to withdraw vs. those who chose continuation, LVEF (30 vs. 30%), LVESVi (88 vs. 83), and LVMi (156 vs. 154) were consistent. All patients were asymptomatic and met the standards of HFimpEF at V1, when echocardiographic parameters and NT-proBNP levels were recorded in all subjects before their final decision was made. Median time between discharge and recovery was 196 days in the withdrawal group vs. 224 days in the continuation group. No statistically significant difference in clinical characteristics was observed between these two final decision groups (Table 1). Mean LVEF was 44% in the withdrawal group vs. 47% in the continuation group.

**Table 1:** Baseline characteristics of the HFimpEF patients.

	**Spironolactone continuation**	**Spironolactone withdrawal**	***P***
	**(*n* = 30)**	**(*n* = 40)**	
Time before recovery (days)	224 (61, 450)	196 (88, 418)	0.59
NT-proBNP (pg/L)	598 (298, 1,609)	721 (371, 1,793)	0.47
Log NT-proBNP concentration	2.85 ± 0.55	2.81 ± 0.63	0.78
**Treatments at recovery, *n* (%)**			
ACE inhibitor or ARB	21 (70)	30 (75)	0.64
β-blocker	28 (93)	39 (98)	0.39
MRA	30 (100)	40 (100)	1.00
Loop diuretic	5 (17)	6 (15)	1.00
ARNI	8 (27)	9 (23)	0.69
CRT/ICD device implantation	1 (3)	2 (5)	0.73
Spironolactone dosage (mg)	19.67 ± 6.69	20.75 ± 7.23	0.53
**Echocardiography parameters at recovery**			
LVEF (%, Simpson)	47 ± 7	44 ± 5	0.11
LVEDD (mm)	61 ± 8	60 ± 7	0.50
LVEDV (ml)	195 ± 57	182 ± 48	0.30
LVEDVI (ml/m^2^)	108 ± 31	102 ± 25	0.37
LVESD (mm)	46 ± 8	46 ± 7	0.90
LVESV (ml)	104 ± 42	100 ± 35	0.66
LVESVi (ml/m^2^)	58 ± 24	56 ± 20	0.73
LVM (g)	240 ± 70	230 ± 79	0.58
LVMi (g/m^2^)	133 ± 37	127 ± 34	0.50

*Data are expressed as mean ± SD, median (interquartile range), or frequency counts (percentages), as appropriate.*

*HFimpEF, heart failure with improved ejection fraction; NT-proBNP, N-terminal pro B-type natriuretic peptide; ACE, angiotensin-converting enzyme; ARB, angiotensin-receptor blocker; MRA, mineralocorticoid receptor antagonist; ARNI, angiotensin receptor neprilysin inhibitor; CRT, cardiac resynchronization therapy; ICD, implantable cardioverter defibrillator; LVEF, left ventricular ejection fraction; LVEDD, left ventricular end-diastolic diameter; LVESD, left ventricular end-systolic diameter; LVEDV, left ventricular end-diastolic volume; LVESV, left ventricular end-systolic volume; LVEDVI, LVEDV indexed by body surface area; LVESVi, LVESV indexed by body surface area; LVMi, left ventricular mass indexed by body surface area*.

### Effects of Spironolactone Withdrawal on Relapse

**Figure 2:**
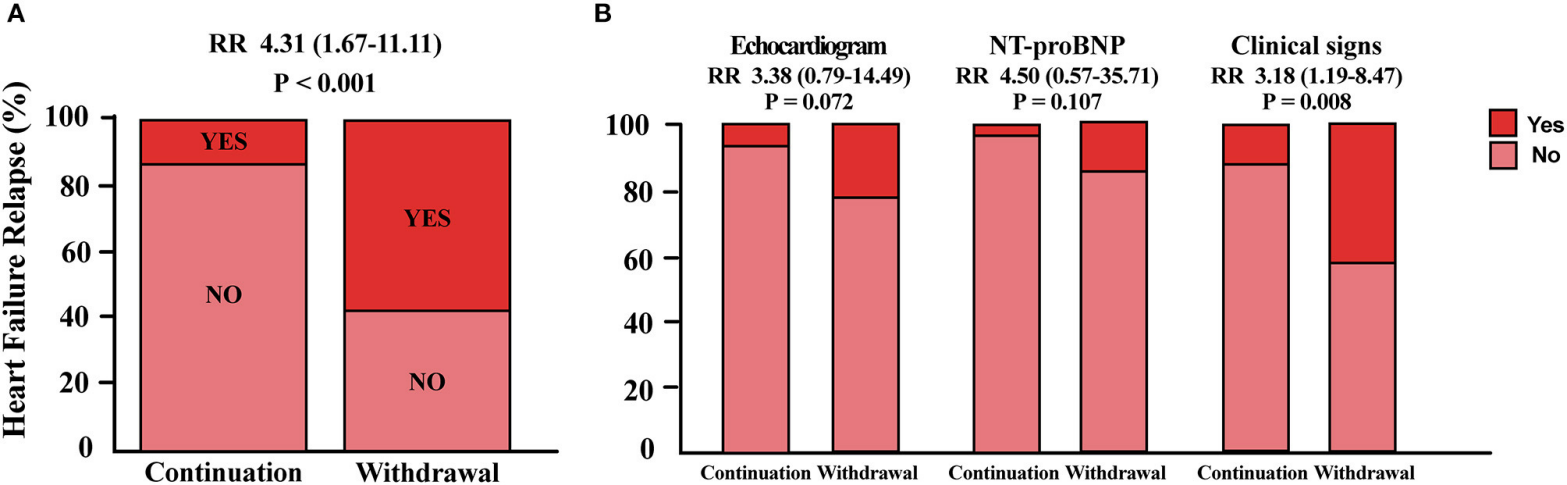
Primary endpoints analysis. **(A)** Proportion of patients who met the primary endpoint in either group. **(B)** Detailed distribution of patients who met one criterion for relapse in each group. RR, relative risk for relapse in the withdrawal group; NT-proBNP, N-terminal pro-B-type natriuretic peptide.

**Figure 3:**
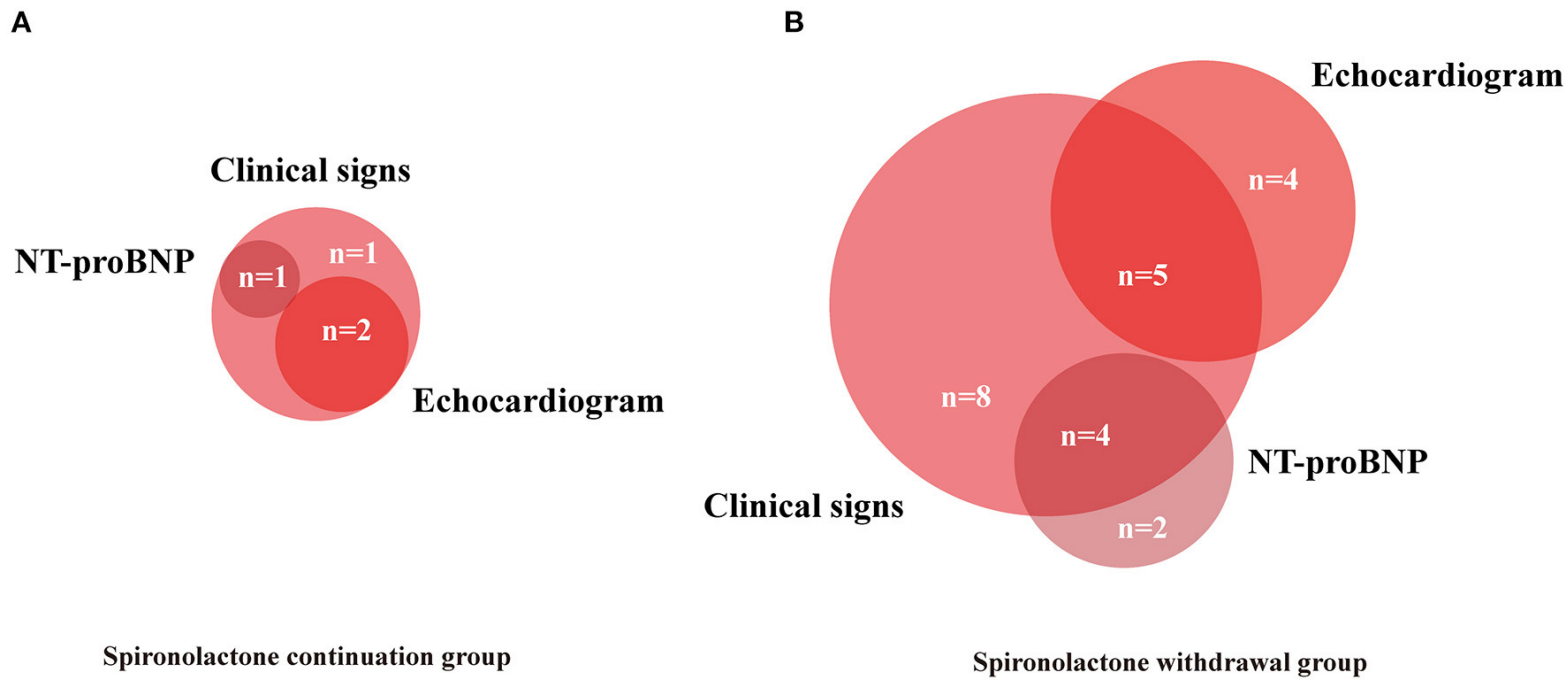
Venn diagram showing components contributing to primary endpoints. Numbers of patients with each combination of endpoints included. **(A,B)** NT-proBNP, N-terminal pro-B-type natriuretic peptide.

For the primary endpoint, 23 patients (58%) in the withdrawal group and 4 patients (13%) in the continuation group met the criteria of relapse. Withdrawal of spironolactone resulted in a relative risk (RR) for relapse of 4.31 (95% CI 1.67–11.11; *p* < 0.001; Figure 2A). In further analysis, it was observed that more patients experienced heart failure symptoms aggravation in the spironolactone withdrawal group (*p* = 0.008) (Figure 2B). In the continuation group, three patients fulfilled more than one criterion of relapse (10%), two (6.7%) met the LVESVi criterion, four (13.3%) presented with heart failure symptoms, and one met the NT-proBNP criterion (Figure 3A). In three out of four subjects with adjudicated signs of relapse, aggravated dyspnea on exertion was recorded. Also, nocturnal paroxysmal dyspnea was presented in the other subject. Of 23 patients who met the primary endpoint in the withdrawal group, 9 fulfilled more than one criterion for relapse (23%), 9 patients (23%) met the LVESVi criterion, 6 (15%) met the NT-proBNP criterion, and 17 (42.5%) had clinical evidence of heart failure (Figure 3B). The major sign of relapse occurred was progressive swelling above the ankle, which accounted for 11 endpoint events. Moreover, four and two subjects experienced exertional dyspnea and nocturnal paroxysmal dyspnea, respectively.

No death, major adverse cardiovascular events, and unplanned cardiovascular hospitalization were reported during the study.

The relative risk of primary outcome was further analyzed in eight subgroups as we have shown in Supplementary Figure 1. No potential heterogeneity of spironolactone clinical decision effect was noticed with a multivariable model that accounted for all potential interactions. Characteristics of patients who subsequently relapsed were compared with those who did not in Table 2. Surprisingly, those who relapsed tend to possess a significantly better LV function at initial diagnosis, as indicated by LVEF and LVESVi.

**Table 2:** Baseline characteristics grouped by endpoint status.

	**Spironolactone continuation group**			**Spironolactone withdrawal group**		
	**Not relapsed**	**Relapsed**	***P***	**Not relapsed**	**Relapsed**	***P***
	**(*n* = 26)**	**(*n* = 4)**		**(*n* = 17)**	**(*n* = 23)**	
Demographics						
Age (years)	55 ± 14	62 ± 10	0.31	50 ± 17	62 ± 15	0.02
Male, *n* (%)	21 (81)	4 (100)	0.34	15 (88)	19 (83)	0.62
Clinical characteristics at initial diagnosis						
Body surface area (m^2^)	1.80 ± 0.27	1.94 ± 0.23	0.34	1.81 ± 0.19	1.79 ± 0.22	0.72
SBP (mmHg)	129 ± 24	137 ± 28	0.57	128 ± 24	134 ± 18	0.34
Diabetes mellitus, *n* (%)	4 (15)	2 (50)	0.11	1 (6)	7 (30)	0.06
Atrial fibrillation, *n* (%)	0 (0)	0 (0)	1.00	1 (6)	1 (4)	1.00
Smoker, *n* (%)	9 (35)	2 (50)	0.55	9 (53)	11 (48)	0.75
NT-proBNP (pg/L)	1,383 (566, 5,894)	3,399 (1,615, 6,433)	0.84	1,275 (782, 5,485)	2,253 (1,294, 3,895)	0.71
Medications at discharge, *n* (%)						
ACE inhibitor or ARB	22 (85)	4 (100)	0.40	15 (88)	21 (91)	0.75
β-blocker	23 (89)	4 (100)	0.47	16 (94)	22 (96)	1.00
MRA	26 (100)	4 (100)	1.00	17 (100)	23 (100)	1.00
Loop diuretic	15 (58)	3 (75)	0.51	8 (47)	11 (48)	1.00
ARNI	3 (12)	0 (4)	0.47	2 (12)	1 (4)	0.38
Echocardiogram at initial diagnosis						
LVEF (%, Simpson)	29 ± 5	33 ± 3	0.17	28 ± 6	32 ± 4	0.01
LVEDD (mm)	67 ± 7	66 ± 5	0.64	68 ± 9	64 ± 6	0.13
LVEDV (ml)	239 ± 58	222 ± 40	0.57	241 ± 66	212 ± 48	0.12
LVEDVi (ml/m^2^)	134 ± 31	117 ± 29	0.30	134 ± 38	119 ± 24	0.15
LVESD (mm)	57 ± 7	53 ± 7	0.35	58 ± 9	52 ± 6	0.02
LVESV (ml)	160 ± 50	138 ± 36	0.42	171 ± 57	132 ± 37	0.02
LVESVi (ml/m^2^)	90 ± 27	73 ± 23	0.25	95 ± 33	74 ± 18	0.01
LVM (g)	282 ± 86	277 ± 95	0.90	293 ± 76	265 ± 96	0.32
LVMi (g/m^2^)	157 ± 42	146 ± 58	0.64	162 ± 41	147 ± 40	0.25

*Data are expressed as mean ± SD, median (interquartile range), or frequency counts (percentages), as appropriate.*

*SBP, systolic blood pressure; NT-proBNP, N-terminal pro B-type natriuretic peptide; ACE, angiotensin-converting enzyme; ARB, angiotensin-receptor blocker; MRA, mineralocorticoid receptor antagonists; ARNI, angiotensin receptor neprilysin inhibitor; LVEF, center ventricular ejection fraction; LVEDD, center ventricular end-diastolic diameter; LVESD, center ventricular end-systolic diameter; LVEDV, center ventricular end-diastolic volume; LVESV, center ventricular end-systolic volume; LVEDVi, LVEDV indexed by body surface area; LVESVi, LVESV indexed by body surface area; LVM, center ventricular mass; LVMi, LVM indexed by body surface area*.

### Impact of Spironolactone on Remodeling

**Figure 4:**
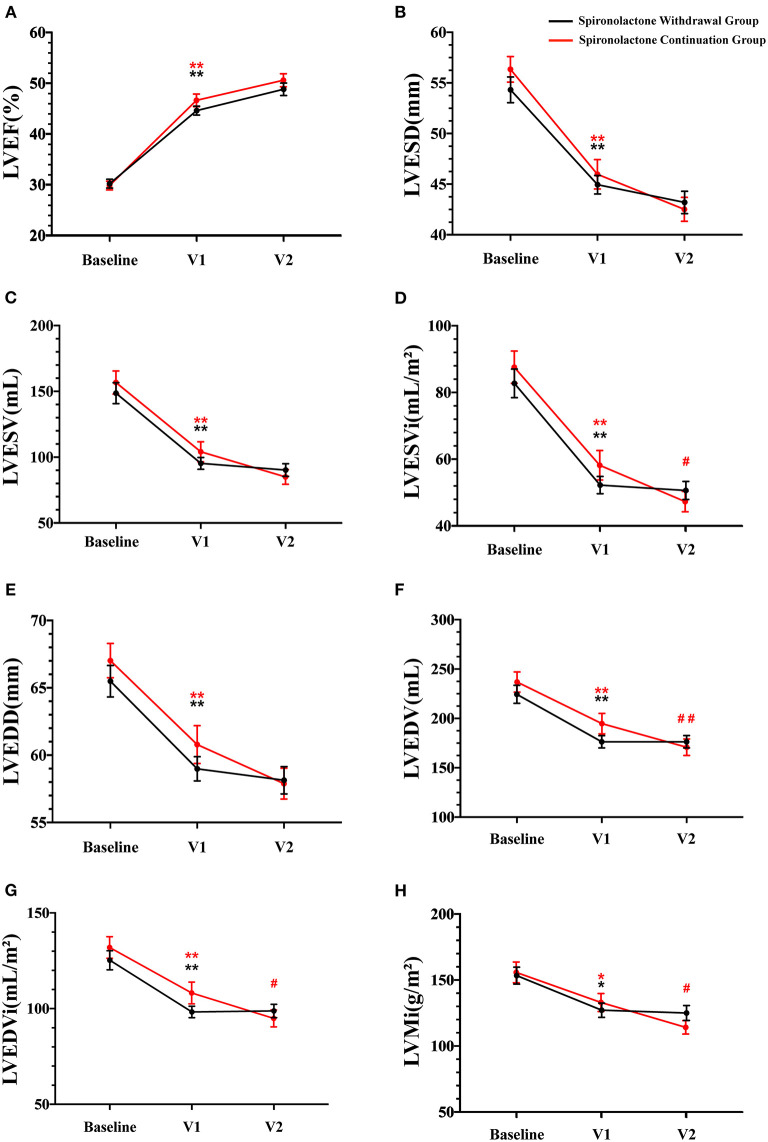
Echocardiographic parameters during follow-up. V1 represented the time when LVEF improved; V2 represented the final 1-year outcome analysis visit; LVEF, left ventricular ejection fraction; LVESD, left ventricular end-systolic diameter; LVESV, left ventricular end-systolic volume; LVESVi, LVESV indexed by body surface area; LVEDD, left ventricular end-diastolic diameter; LVEDV, left ventricular end-diastolic volume; LVEDVi, LVEDV indexed by body surface area; LVMi, left ventricular mass indexed by body surface area. Data are represented as the mean ± SEM. *p < 0.05 and **p < 0.01 compared to values at the time of first diagnosis; ^#^*p* < 0.05 and ^##^*p* < 0.01 compared to values at the time of improved ejection fraction identified.

Changes in echocardiography parameters were assessed at the time of first diagnosis, the time of improved ejection fraction identified (V1), and 1-year clinic visit after recovery (V2). There was a persistent downtrend in LVESVi, LVEDV, LVEDVi, and LVMi with spironolactone continuation (*p* < 0.05, Figures 4D,F–H), while no significant difference between V1 and V2 was detected in LVEF, LVEDD, LVESD, and LVESV (Figures 4A–C,E). Worth noticing, the downtrend mentioned earlier was not preserved in the spironolactone withdrawal group.

Regarding the effect of spironolactone withdrawal in HFimpEF patients on ventricular remodeling, a significant higher LVMi were observed at 1-year final analysis compared with the continuation group. As our cohort reflected, although the consequence was not statistically significant in other parameters, including LVEF and LVESVi, a nominally detrimental trend of heart remodeling in the withdrawal group was noticed (Table 3). NT-proBNP, as an objective serum biomarker for ventricular stress and neurohormonal activation, did not show critical intergroup difference after log transformation (2.05 vs. 2.58) at V2 in this study. However, a larger numerical reduction within a year was detected in the spironolactone continuation cohort.

**Table 3:** Echocardiographic parameters over a 1-year follow-up period.

	**Spironolactone continuation (*n* = 30)**	**Spironolactone withdrawal (*n* = 40)**	***P*** **(ANCOVA)**
**LVEF, %**			0.23
Visit 1	46 ± 7	45 ± 6	
Visit 2	51 ± 7	49 ± 8	
**LVEDD, mm**			0.56
Visit 1	61 ± 8	59 ± 6	
Visit 2	58 ± 6	58 ± 6	
**LVESD, mm**			0.91
Visit 1	46 ± 8	45 ± 6	
Visit 2	43 ± 6	43 ± 7	
**LVEDV, ml**			0.52
Visit 1	195 ± 57	176 ± 39	
Visit 2	171 ± 46	176 ± 40	
**LVESV, ml**			0.82
Visit 1	104 ± 42	95 ± 28	
Visit 2	85 ± 31	90 ± 30	
**LVEDVi, ml/m** ^**2**^			0.57
Visit 1	108 ± 31	98 ± 19	
Visit 2	95 ±24	99 ± 22	
**LVESVi, ml/m** ^**2**^			0.77
Visit 1	58 ± 24	52 ± 16	
Visit 2	47 ± 17	51 ± 17	
**LVM, g**			0.08
Visit 1	240 ± 70	230 ± 79	
Visit 2	206 ± 53	225 ± 78	
**LVMi, g/m** ^**2**^			0.04
Visit 1	133 ± 37	127 ± 34	
Visit 2	114 ± 28	125 ± 36	
**Log NT-proBNP concentration, pg/L**			0.09
Visit 1	2.85 ± 0.55	2.81 ± 0.63	
Visit 2	2.05 ± 0.61	2.58 ± 0.83	

*Data are expressed as mean ± SD. Difference of each echocardiographic parameter at visit 2 was analyzed using regression analysis of covariance (ANCOVA) adjusted for the measurement at visit 1.*

*LVEF, left ventricular ejection fraction; LVEDD, left ventricular end-diastolic diameter; LVESD, left ventricular end-systolic diameter; LVEDV, left ventricular end-diastolic volume; LVESV, left ventricular end-systolic volume; LVEDVi, LVEDV indexed by body surface area; LVESVi, LVESV indexed by body surface area; LVM, left ventricular mass; LVMi, LVM indexed by body surface area; NT-proBNP, N-terminal pro-B-type natriuretic peptide*.

## Discussion

MRAs are recognized as an integral part of the pharmacological treatment of HFrEF according to the contemporary standard of care (11, 12). This class of drugs has demonstrated beneficial effects on morbidity and mortality in HFrEF patients (13–15). Although there is substantial evidence supporting use of MRAs for chronic HFrEF management, its benefits for the outcomes after LVEF recovery are less clear. Our focus on discontinuation of MRA could be rationalized by previous randomized trials (16, 17), showing MRAs have not demonstrated additional effects on LV functions when applied in addition to other optimal background therapy. To the best of our knowledge, this is the first study to investigate withdrawal of spironolactone alone in patients with recovered dilated cardiomyopathy. Our cohort study showed that spironolactone withdrawal was accompanied with a higher rate of relapse in dilated cardiomyopathy with improved ejection fraction. This finding suggests that MRA therapy retainment is necessary to maintain myocardial remission and should not be discontinued routinely in patients with clinically recovered heart function. The sustained benefit of spironolactone could be attributed to MRAs' favorable effects on collagen metabolism in all stages of heart failure. As the recent HOMAGE trial demonstrated, MRAs reduced synthesis serum marker of type-I collagen, while they increased the degradation marker even in a population of structural heart disease with few or no symptoms of heart failure (18).

Further analyses of individuals who met the primary endpoint in the withdrawal group revealed that 74% emerged clinical evidence of heart failure, while no substantial LVEF reduction was observed. These results could be partially explained by the pharmacological mechanism of MRAs, which not only block aldosterone receptor in the heart but also act as a diuretic (19). Withdrawing MRAs may increase patients' susceptibility to heart failure signs and symptoms attributed to potential fluid retention. Since the calculation of LVEF integrates LV end-diastolic volume in the denominator of the equation, nominal reduction in LVEF may lag behind LVEDV (20). Potentially, more reduction in LVEF or unplanned hospital admission would occur if a longer period of spironolactone withdrawal proceeded. However, when early signs of deterioration were noticed, a rapid response was ensured in this cohort, for patient safety concern, including resuming spironolactone treatment and any other necessary intervention before such decompensation aggravated. Therefore, increase in LVESVi for more than 15% was chosen as a primary endpoint. As previously adopted by other randomized trials (21, 22), both cardiac contractility and negative remodeling traits could be sensitively reflected by LVESVi, which is also influenced less by loading conditions.

According to our study, sustained benefits were observed with MRA therapy after the LVEF recovered. However, in the continuation group, 13% of patients still suffered from a relapse of heart failure during the follow-up. These results indicated that cardiac function recovery after optimal medicine therapy does not reflect a full and stable myocardial recovery but rather a remission, which may recur under certain circumstances (23, 24). The ability to precisely distinguish between patients who achieved complete recovery and those who only achieved remission is a cardinal goal. Conventional clinical assessment methods including echocardiogram and NT-proBNP at baseline cannot effectively differentiate these two phenotypes, as Table 3 depicted. Patients who relapsed later contrarily demonstrated better echocardiographic parameters at baseline, indicating novel pathologic pathways may be involved in the recovery course. Recognition of specified gene variants or serum biomarkers could act as a tool to stratify the risk of heart failure relapse (25).

We also conducted a subgroup analysis in hope of identifying patients with least relapse risk after spironolactone withdrawal. Since both physicians and patients prefer a less complex medical therapy, compliance should be enhanced to avoid side-effects. Our results re-emphasized that there was no safe discontinuation of spironolactone in patients with idiopathic dilated cardiomyopathy with improved ejection fraction in any subgroups. Even in patients with lower NT-proBNP or higher LVEF at either baseline or follow-up, a safe spironolactone withdrawal cannot be promised.

Most contemporary studies focusing on treatment withdrawal in patients with recovered dilated cardiomyopathy have been retrospective, and results were controversial (26–28). To date, the open-label, single-center TRED-HF (Withdrawal of Pharmacological Treatment for Heart Failure in Patients with Recovered Dilated Cardiomyopathy) trial was the only pilot randomized trial investigating the impact of treatment withdrawal for recovered dilated cardiomyopathy, which concluded that treatment should continue indefinitely (29). Despite existing preliminary clinical evidence, patients taking multiple pills daily still frequently ask for at least partial medication cessation, especially after their recovery. Lack of affirmatory medical guidelines could affect patient compliance and undermine fragile stability. Different from the phased withdrawal of all heart failure medications adopted in the TRED-HF trial, including diuretics, MRAs, β-blockers, and RAAS inhibitors, our study explored whether spironolactone could be specifically withdrawn safely after a clinical recovery. Also, our real-world cohort was composed of patients with lower LVEF and all prescribed MRAs at baseline, which could represent a population recovered from more severe dilated cardiomyopathy and responded well to treatment. As the exploratory analysis in TRED-HF suggested, prescription of an MRA before withdrawal may be associated with higher risk of disease relapse. Consistent with previous suggestion, our data re-emphasized not even spironolactone alone should be withdrawn after LVEF improved in dilated cardiomyopathy patients.

Several limitations persisted in the study. Since this was an observational, open-label, non-randomized, monocentric pilot design with a small number of patients recruited and soft endpoints adjudicated by clinicians, which increased the risk of bias, the power to examine the association between drug withdrawal and relapse was limited. To further elaborate this critical clinically relevant topic, a double-blind, multiple-centered, randomized trial is now in preparation. Moreover, our study focused on patients with idiopathic dilated cardiomyopathy, so the results should be interpreted within this population. For patients with improved left ventricular function secondary to ischemic or hypertensive cause, the best practice remains to be determined.

## Conclusion

In this prospective observational cohort study, withdrawal of spironolactone treatment in dilated cardiomyopathy patients with improved ejection fraction resulted in more disease relapses, and such discontinuation should not be routinely attempted in these patients. Therefore, spironolactone withdrawal might not be a safe strategy for a selected subgroup of LVEF improved patients in the outpatient setting.

## Data Availability Statement

The raw data supporting the conclusions of this article will be made available by the authors, without undue reservation.

## Ethics Statement

The studies involving human participants were reviewed and approved by Institutional Review Board of Ruijin Hospital, Shanghai Jiao Tong University School of Medicine. The patients/participants provided their written informed consent to participate in this study.

## Author Contributions

WJ designed and supervised the study. YC, ZQ, JJ, XS, FH, and JT performed data collection. YC, ZQ, and JJ performed data analysis. YC, ZQ, and FH interpreted the results of the analysis. YC and ZQ drafted the article. All authors read and contributed to the article.

## Funding

This study has received funding from National Natural Science Foundation of China (81970337).

## Conflict of Interest

The authors declare that the research was conducted in the absence of any commercial or financial relationships that could be construed as a potential conflict of interest.

## Publisher's Note

All claims expressed in this article are solely those of the authors and do not necessarily represent those of their affiliated organizations, or those of the publisher, the editors and the reviewers. Any product that may be evaluated in this article, or claim that may be made by its manufacturer, is not guaranteed or endorsed by the publisher.
