# Supplementary material for: Outcomes of Spironolactone Withdrawal in Dilated Cardiomyopathy With Improved Ejection Fraction
Source: Front Cardiovasc Med. 2021 Sep 16;8:725399. doi: 10.3389/fcvm.2021.725399 (PMC8481596; doi:10.3389/fcvm.2021.725399)
Supplement: Supplementary file 1 [file Data_Sheet_1.PDF]

Supplementary Table 1. Baseline characteristics at initial diagnosis

	Spironolactone continuation (n=30)	Spironolactone withdrawal (n=40)	P value
Demographics			
Age (years)	56 ± 13	57 ± 16	0.77
Male, n (%)	25 (83)	34 (85)	0.85
Clinical characteristics at initial diagnosis			
Body surface area (m^2)	1.81 ± 0.27	1.80 ± 0.20	0.77
SBP (mmHg)	130 ± 24	131 ± 21	0.85
Diabetes mellitus, n (%)	6 (20)	8 (20)	1.00
Atrial fibrillation, n (%)	0 (0)	2 (5)	0.21
Smoker, n (%)	11 (37)	20 (50)	0.27
NYHA Class IV, n (%)	6 (20)	8 (20)	1.00
NT-proBNP (pg/L)	1727 (940, 5894)	2169 (971, 4310)	0.90
QRS duration (ms)	108 ± 24	110 ± 26	0.80
CMR with LGE, n (%)	9 (30)	9 (23)	0.48
Family history, n (%)	0 (0)	0 (0)	1.00
Medications at discharge, n (%)			
ACE inhibitor or ARB	26 (87)	36 (90)	0.66
β-blocker	27 (90)	38 (95)	0.42
MRA	30 (100)	40 (100)	1.00
Loop diuretic	18 (60)	19 (48)	0.30
ARNI	3 (10)	3 (8)	0.71
Echocardiogram at initial diagnosis			
LVEF (%, Simpson)	30 ± 5	30 ± 6	0.69
LVEDD (mm)	67 ± 7	65 ± 7	0.37
LVEDV (mL)	237 ± 56	224 ± 57	0.37
LVEDVi (mL/m²)	132 ± 31	125 ± 32	0.38
LVESD (mm)	56 ± 7	54 ± 8	0.28
LVESV (mL)	157 ± 48	149 ± 50	0.50
LVESVi (mL/m²)	88 ± 27	83 ± 27	0.46
LVM (g)	282 ± 85	277 ± 88	0.82
LVMi (g/m²)	156 ± 43	154 ± 40	0.80

Data are expressed as mean ± SD, median (interquartile range), or frequency counts (percentages), as appropriate. SBP, systolic blood pressure; NYHA, New York Heart

Association; NT-proBNP, N-terminal pro B-type natriuretic peptide; CMR, cardiac magnetic resonance; LGE, late gadolinium enhancement; ACE, angiotensin-converting enzyme; ARB, angiotensin-receptor blocker; MRA, mineralocorticoid receptor antagonists; ARNI, Angiotensin receptor neprilysin inhibitor; LVEF, left ventricular ejection fraction; LVEDD, left ventricular end-diastolic diameter; LVESD, left ventricular end-systolic diameter; LVEDV, left ventricular end-diastolic volume; LVESV, left ventricular end-systolic volume; LVEDVi, LVEDV indexed by body surface area; LVESVi, LVESV indexed by body surface area; LVM, left ventricular mass; LVMi, LVM indexed by body surface area.

Subgroup	Continuation	Withdrawal	Relative Risk (95%CI)
**All Patients**	**30**	**40**	**0.23(0.09–0.60)**
**Age (yrs)**			
≥ 65	6	15	0.23(0.04–1.40)
< 65	24	25	0.26(0.08–0.81)
**Gender**			
Male	25	34	0.22(0.07–0.65)
Female	5	6	0.30(0.05–1.89)
**History of Diabetes**			
Yes	6	8	0.38(0.12–1.22)
No	24	32	0.17(0.04–0.66)
**SBP at Baseline**			
> Median (128 mmHg)	13	21	0.27(0.07–1.02)
≤ Median (128 mmHg)	17	19	0.20(0.05–0.79)
**LVEF at Baseline**			
> Median (32%)	12	23	0.21(0.06–0.77)
≤ Median (32%)	18	17	0.38(0.08–1.69)
**NT–proBNP at Baseline**			
> Median (2019 pg/mL)	14	21	0.23(0.06–0.87)
≤ Median (2019 pg/mL)	16	19	0.24(0.06–0.93)
**LVEF after Recovery**			
> Median (44%)	18	17	0.21(0.05–0.84)
≤ Median (44%)	12	23	0.27(0.07–1.01)
**NT–proBNP after Recovery**			
> Median (672 pg/mL)	14	21	0.20(0.05–0.74)
≤ Median (672 pg/mL)	16	19	0.30(0.07–1.20)

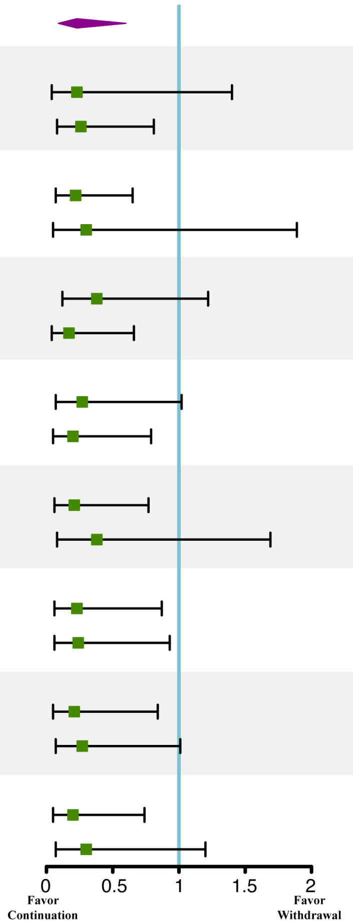

Supplementary Figure 1. Primary Outcome in Subgroups. The primary outcome was relapse of dilated cardiomyopathy within 12 months. The diamond represents the overall effect and arrows indicate the upper or lower boundary of the confidence interval is off the scale. SBP, systolic blood pressure; NT-proBNP, N-terminal pro-B-type natriuretic peptide; LVEF, left ventricular ejection fraction.
